# The complete chloroplast genome sequence of *Huperzia javanica* (sw.) C. Y. Yang in Lycopodiaceae

**DOI:** 10.1080/23802359.2017.1310603

**Published:** 2017-04-09

**Authors:** Hong-rui Zhang, Jong-Soo Kang, Ronald L. L. Viane, Xian-Chun Zhang

**Affiliations:** a State Key Laboratory of Systematic and Evolutionary Botany, Institute of Botany, Chinese Academy of Sciences, Beijing, China;; b University of Chinese Academy of Sciences, Beijing, China;; c Research Group Pteridology, Department of Biology, Ghent University, Gent, Belgium

**Keywords:** *Huperzia javanica*, *Huperzia*, Lycopodiaceae, chloroplast genome

## Abstract

*Huperzia javanica* (Sw.) C. Y. Yang is a valuable medical herb used for treating Alzheimer’s disease. Here, we described the complete chloroplast genome of *H. javanica* using Illumina paired-end sequencing. The total genome length is 154,415 bp, containing 119 unique genes, with 86 protein-coding genes, 29 tRNA genes, and 4 rRNA genes. The gene content and their order are consistent with two previously reported *Huperzia* genomes. The overall GC content of the chloroplast genome of *H. javanica* is 36.4%. The topology of our maximum-likelihood tree is consistent with topologies found in previous studies, with *H. javanica* sister to a clade of *H. serrata* and *H. lucidula*. We support the recognition of *H. javanica* as an independent species. *Huperzia serrata* is more closely related to *H. lucidula* than to *H. javanica*.

*Huperzia javanica* C. Y. Yang (Lycopodiaceae) is a perennial, evergreen, South East Asian herb found in South China, India, Japan, South Korea, and the Philippines (Sun 2015). It grows on shady places in humid forests and valleys at an elevation ranging from 300–1200 m (Shrestha & Zhang 2015; Sun 2015). *Huperzia* species are known as valuable medicinal herbs. Especially, *H. javanica* and *H. serrata* are used for the treatment of Alzheimer’s disease (Tang 1996; Wang et al. 1998; Yang et al. 2008). Recently, *H. javanica* was distinguished as independent species from *H. serrata* complex through a previous study using morphological and climatic data (Shrestha & Zhang 2015), but we could not distinguish these species on the dried condition for making the oriental medicine based on only morphology. Chloroplast genomes are widely used in molecular phylogeny, to assess genetic diversity in conservation genetics, and in molecular identification such as DNA barcoding (Burke et al. 2012; Huang et al. 2014; Walker et al. 2014). In this study, we describe the chloroplast genome of *H. javanica* and reveal the phylogenetic relationship between *H. javanica* and *H. serrata*, thus contributing to the molecular identification and the conservation biology of *H. javanica*. The annotated sequence was deposited in GenBank under accession number KY609860.

Leaf material of *H. javanica* was collected in Shaoguan, Guangdong province in south China. The voucher specimen (*R. Wei CBL011*) has been deposited in the Herbarium of the Institute of Botany, Chinese Academy of Sciences (PE). Total DNA was extracted from silica gel dried leaves with a modified cetyltrimethylammonium bromide (CTAB) method (Li et al. 2013). The NEBNext DNA Library Prep Kit for Illumina (New England Biolabs, Ipswich, MA) was used for library construction. Paired-end reads of 2 × 150 bp then were generated using an Illumina HiSeq PE150 (Illumina, San Diego, CA). A total of 8,688,705 paired-end sequence reads of 150 bp were generated, of which 230,793 paired-ends reads belong to the chloroplast genome. The chloroplast genome data were extracted using *H. lucidula* (AY660566) as a reference and assembled *de novo* with Geneious v. R9.0.5 (Kearse et al. 2012). The initial annotation of *H. javanica* chloroplast genome was performed using Dual Organellar GenoMe Annotator (DOGMA; Wyman et al. 2004). After initial annotation, the putative starts, stops, and intron positions were determined by comparison with homologous genes in previously reported *Huperzia* chloroplast genomes. The tRNA genes were annotated using DOGMA and tRNAscan-SE (Schattner et al. 2005). The circular chloroplast genome map was drawn using the OGDraw program (Lohse et al. 2013).

The complete size of the *H. javanica* chloroplast genome is 154,415 bp, which includes a pair of inverted repeat (IR) regions of 15,315 bp separated by a small single copy (SSC) region of 19,667 bp and a large single copy (LSC) region of 104,120 bp similar to the previously reported *Huperzia* chloroplast genomes (Wolf et al. 2005; Guo et al. 2016). The *H. javanica* chloroplast genome contains 119 genes, 11 of which are duplicated in the IR region, giving a total of 130 genes. The chloroplast genome of *H. javanica* contains 29 distinct tRNAs, four of which are duplicated in the IR region. 13 genes (*atpF*, *ndhA*, *ndhB*, *petB*, *petD*, *rps12*, *rps16*, *rpl2*, *rpl16*, *rpoC1*, *trnA*-*UGC*, *trnL*-*UAA*, *trnV*-*UAC*) contain one intron, while two genes (*clpP*, *ycf3*) contain two introns. The *rps12* gene is divided in two independent transcription units. The overall GC content is 36.4%, in particular 34.5% in the LSC, 32.9% in the IR, and 45.0% in the SSC region.

Phylogenetic analysis based on 72 protein-coding genes was performed using extra species from Lycopodiaceae to Cyatheales in the PPG I system (PPG I 2016), and two Bryophyte species (*Anthoceros angustus* and *Marchantia polymorpha*) as the outgroup (Figure 1). A total of 55,034 bp was aligned using MAFFT (Katoh et al. 2002). Maximum-likelihood (ML) analysis was performed using RAxML v. 7.4.2 with 1000 bootstrap replicates and the GTR + I + G model (Stamatakis 2006; Darriba et al. 2012). Our ML tree topology is consistent with topologies published in previous studies (Lu et al. 2015; PPG I 2016) (Figure 1). Although *H. javanica* is morphologically more similar to *H. serrata*, the phylogenetic relationship of *H. serrata* is closer to that of *H. lucidula* than to that of *H. javanica* (Figure 1). Moreover, this is confirmed by the sequence variation rates of the data matrix of 72 aligned protein-coding gene sequences of these three *Huperzia* species, which show that the sequence variation rate between *H. serrata* and *H. javanica* (0.411%) is slightly higher than that between *H. serrata* and *H. lucidula* (0.099%). These results are consistent with those of a previous study using morphological and climatic data, in which *H. javanica* was separated from *H. serrata* and treated as independent species (Shrestha & Zhang 2015).

**Figure 1.**
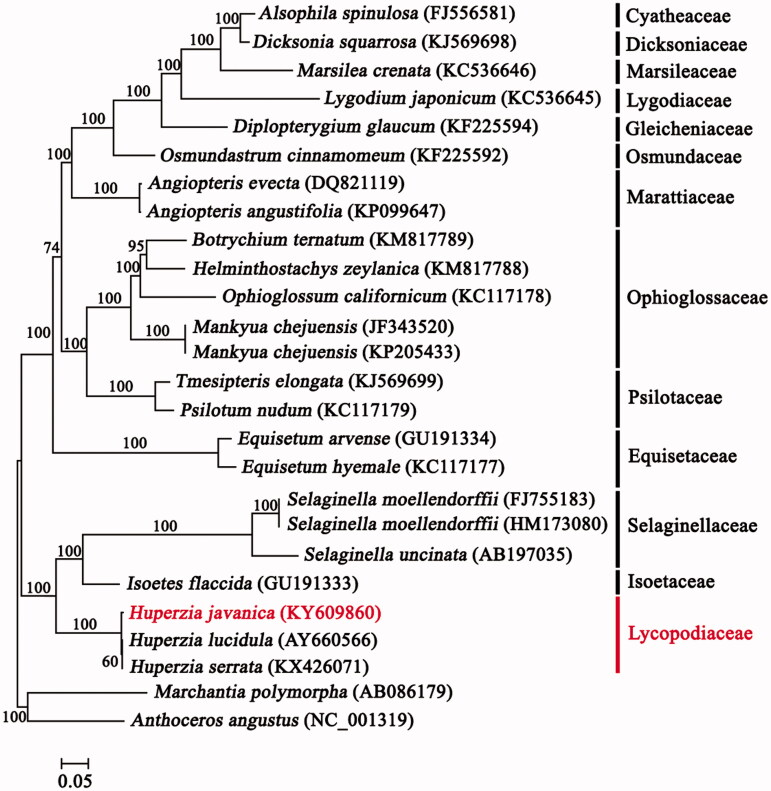
Phylogenetic tree based on 72 protein-coding genes using the ML method. Taxon in bold is the new genome reported in this study. Bootstrap values are shown above the nodes.
